# Pyloric Tuberculosis and Tubercular Abscess of Stomach

**Published:** 1914-08

**Authors:** 


**Pyloric Tuberculosis and Tubercular Abscess of Stomach.** Case report by H. Schlesinger, Muench. Med. Woch., page 987, 1914.

